# Multi-Dimension and Multi-Feature Hybrid Learning Network for Classifying the Sub Pathological Type of Lung Nodules through LDCT

**DOI:** 10.3390/s21082734

**Published:** 2021-04-13

**Authors:** Jiacheng Fan, Jianying Bao, Jianlin Xu, Jinqiu Mo

**Affiliations:** 1 School of Mechanical Engineering, Shanghai Jiao Tong University, Shanghai 200240, China; fan_jiacheng@sjtu.edu.cn (J.F.); agnes_0130@sjtu.edu.cn (J.B.); 2 Department of Pulmonary, Shanghai Chest Hospital, Shanghai Jiao Tong University, Shanghai 200240, China; xujianlin1018@163.com

**Keywords:** neural network, low-dose spiral computed tomography (LDCT), lung nodules, sub pathological types classification

## Abstract

In order to develop appropriate treatment and rehabilitation plans with regard to different subpathological types (PILs and IAs) of lung nodules, it is important to diagnose them through low-dose spiral computed tomography (LDCT) during routine screening before surgery. Based on the characteristics of different subpathological lung nodules expressed from LDCT images, we propose a multi-dimension and multi-feature hybrid learning neural network in this paper. Our network consists of a 2D network part and a 3D network part. The feature vectors extracted from the 2D network and 3D network are further learned by XGBoost. Through this formation, the network can better integrate the feature information from the 2D and 3D networks. The main learning block of the network is a residual block combined with attention mechanism. This learning block enables the network to learn better from multiple features and pay more attention to the key feature map among all the feature maps in different channels. We conduct experiments on our dataset collected from a cooperating hospital. The results show that the accuracy, sensitivity and specificity of our network are 83%, 86%, 80%, respectively It is feasible to use this network to classify the subpathological type of lung nodule through routine screening.

## 1. Introduction

Lung cancer is one of the major cancers that causes serious harm to human health. With the growth of the smoking population and the deterioration of the atmospheric environment in recent years, the morbidity and mortality rates of lung cancer remain high [1]. Lung nodules, round or irregular lesions, are the symbol of lung cancer. The nodules are classified as benign nodules and malignant nodules. Malignant nodules are cancerous and can be further divided by decreasing degree of malignancy into invasive adenocarcinoma (IA), minimally invasive adenocarcinoma (MIA), adenocarcinoma in situ (AIS), and atypical adenomatous hyperplasias (AAH) [2]. Among them, MIA, AIS and AAH are relatively early stages of lung cancer. They are regarded as preinvasive lesions (PILs) because their disease-specific survival rates are almost the same. Clinically a subpathological lesion diagnosis is equivalent to a diagnosis of PIL and IA.

Early diagnosis of the malignant lung nodules’ subpathological type are important for treatment and rehabilitation planning. On the one hand, there are almost no symptoms [3] in the early stages of lung cancer compared with other cancers. Statistics show that the 5-year survival rate of patients with PILs after surgery is almost 90−100% [4]. Correspondingly, the 5-year survival rate of patients with IAs is much lower. If the subpathological type of lung nodules could be identified early, it could prevent the lesions from deteriorating. The surgical treatment of patients with PILs is relatively conservative, whereas the surgical treatment of patients with IAs is quite different. Generally speaking, the diagnosis of the malignant lung nodules’ subpathological type should through frozen sections taken during the surgery. In this case, doctors should wait for the frozen section results to determine the surgical treatment accordingly, which prolongs the duration of the operation. Ideally, it would be better to determine the subpathological type of malignant lung nodules before surgery through non-surgical CT images, but a main obstacle is that even experienced doctors have difficulty in classifying them from CT images. If there were a way of diagnosing lung cancer in the early stage through CT images, it would benefit doctors and patients in many ways.

It can be concluded from the background that doctors make treatment plans with regard to the subpathological type. Also, if the early stage of lung cancer (PILs) can be diagnosed, the patient will have a better chance of recovery. Therefore, there is a demand for PILs to be diagnosed through routine screening. Since low-dose spiral computed tomography (LDCT) [5] is commonly used for routine medical examinations provided by agencies, it is the proper diagnostic tool.

During these years, with the development of computer science, deep learning methods are considered a promising way of assisting the diagnosis of diseases [6] through CT or other medical images. These methods train neural networks using large amounts of CT or other medical images. The dedicated designed neural networks can learn key features of how to diagnose a disease. Then in practical application, neural networks are able to give the diagnosis results for a given image. Compared with diagnoses by doctors, deep learning methods are advantageous because these methods are not subject to human factors such as subjectivity, experience difference and fatigue [7]. After years of study and application, deep learning methods have proved their effectiveness and superiority regarding diagnosis based on CT or other medical images [8].

With respect to the diagnosis of lung cancer using deep learning, there are many studies. Shen et al. [9] proposed a multi-scale CNN (MSCNN). They trained the network with three scales of the input image. The network is a standard shallow CNN which can hardly represent the features clearly. They further improved MCNN and developed multi-crop CNN (MC-CNN) [10]. This network substitutes standard max-pooling layer with their designed multi-crop pooling layer to extract multi-scale features. Al-Shabi et al. [11] presented a network combining blocks extracting local features and blocks extracting global features. Xu et al. [12] proposed a MSCS-DeepLN network. The main contribution of this network is a light model ensemble net. It can extract features of different scale. They also generalize a new AUC surrogate loss to solve the category imbalance problem. Li et al. [13] proposed a fusion network that combines handcrafted features (HF) into the features learned at the output layer of a 3D deep convolutional neural network (CNN). Sri Varun et al. [14] embedded six external shape-based features into the convolutional neural network. Their work provides the medical classification task with a new idea of learning from external feature maps. However, they didn’t consider the feature expressed in 3D. El-Regaily et al. [15] proposed a multi-view CNN to increase the accuracy of classification. They trained the network with axial, coronal, and sagittal views obtained from the 3D model of nodules. Ni et al. [16] designed an automatic diagnosis network for finding the location of the lung nodules and classifying the invasiveness of the nodule at the same time, but their work is based on HRCT images which are not suitable for routine screening. Most recently, Lyu et al. [17] designed ML-xResNet to classify the malignancy of nodules. It is constructed by three-level parallel ResNets with different convolution kernel sizes. The residuals are connected not only with the current level but also with other levels in a crossover manner. The main drawback of [17] is that they do not consider the features in 3D. Additionally, there are some researches focused on the localization of the lung nodules such as [18,19]. They designed their method based on the idea of transfer learning so that the training process will be compressed greatly. Apart from transfer learning, there is also the reinforcement learning method [20] that provide a new way of solving the location detection problem. These localization researches can better improve the whole process of computer diagnosis and treatment.

**Figure 1:**
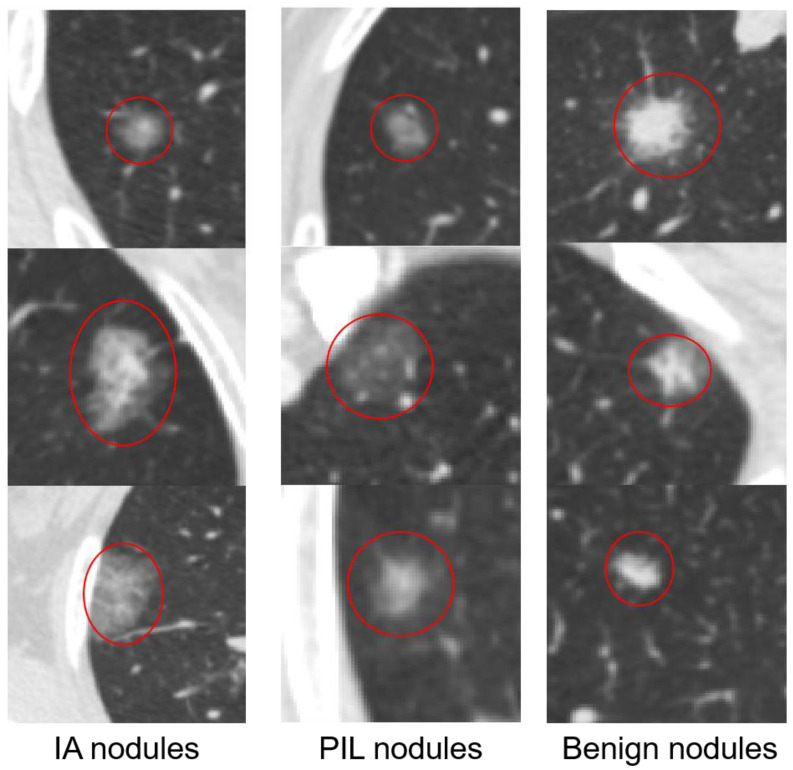
Example of PIL and IA image.

Existing studies are thus not suitable for our classification task. For one thing, most of the existing studies focus on the classification of benign nodules and malignant nodules based on LIDC-IDRI dataset. They didn’t address the classification problem of sub pathological types through LDCT images. For another, the network architecture of the existing studies either consider multi-feature or multi dimension. They have not learned from multi-feature and multi dimension at the same time even if they all express features. Our paper aims to classify the subpathological type of lung nodules through LDCT. Different from the classification of benign nodules and malignant nodules, the classification of PILs and IAs has its own characteristic. The density of the nodules is small and they express few features regarding the area adjacent to the nodules. As shown in Figure 1, the difference between IAs and PILs are very similar. The low resolution images obtained from LDCT further aggravate the problem of information loss.

In this paper, we propose a multi-level and multi feature hybrid learning model. This model is designed to be composed of a 2D network part and a 3D network part so that the network can learn from different dimension. Based on the features of the raw images, we design a learning block with attention mechanism [21] on the basis of ResNet [22]. The attention mechanism in the block can give different weights to different feature channels, so the learning block can better learn from multi-feature and resolve the poor information problem. After training the 2D network part and 3D network part, the final feature vectors extracted from the 2D network part and 3D network part are combined and further learned by XGBoost [23]. It can better predict the results through establishing decision trees. Our work contributes in two ways. In the aspect of network modeling, the idea of fusing different features from different dimensions may help with the design of other CT-related diagnostic researches. The whole formation enables the network with a more powerful ability in classifying images without unique features. In the aspect of diagnosis practices, the output result of our network can help with the doctors diagnose and make treatment plans before surgery.

## 2. Materials and Methods

### 2.1. Dataset

We collected 1752 cases from the Shanghai Chest Hospital. Among these cases, there were 737 benign nodule cases, 339 IA nodule cases and 676 PIL nodule cases. In each case, there are many CT images. The nodules position in CT images are labeled by doctors in the form of rectangles. The center CT image of all the CT images which contain the nodules in a case is also labeled by doctors as the reference center CT image. One unique characteristic of our dataset is that all these cases in our dataset were diagnosed by biopsy which is the golden standard of lung cancer diagnosis. As for commonly used LIDC-IDRI dataset [24], there is no golden standard of malignancy diagnosis in the database.

Based on the analysis of the dataset, the features of lung nodule can be observed from planar space in the CT image and spatial space in the stacked CT images. Therefore, we construct 2D samples and 3D samples from CT images of each case to represent the features of lung nodule.

#### 2.1.1. 2D Samples

We extract three maps as 2D samples in image planar space. The first map is the original CT image of the lung nodule. This image is cropped from the original CT image. If the upper left coordinate of the manual labeled nodule rectangle is $\left(i_{L},j_{L}\right)$ and the lower right coordinate of the manual labeled nodule rectangle is $\left(i_{R},j_{R}\right)$, the center point of the manual labeled nodule rectangle is $\left(i_{L}+\frac{i_{R}}{2},j_{L}+\frac{j_{R}}{2}\right)$. We take this center point as the center point of the first map and clip the CT image with a pixel block size of 32 × 32.

The second map is a texture feature map of the original CT image. We employ Local Binary Pattern (LBP) [25] on the original CT image to formulate texture feature map. Compared with other feature operators, LBP is enough for expressing the feature of the image. If the operator is more complicated, the network may not well understand the feature map. LBP operator can express the local features of the image well and has the advantages of gray invariance and rotation invariance. 

The third map is edge feature map of the original CT image. The characteristics of the nodule edge and the lobular edge play an important role in indicating whether the nodule is malignant or not. Edge feature map is 0–1 map. It contains less information compared with the first map and the second map. In order to balance the information of different, we use Canny operator [26] to extract the edge feature map of the original CT image in this paper for it can extract as many edges as possible.

#### 2.1.2. 3D Samples

In view of the fact that the CT images are acquired in 1 mm intervals on the plane perpendicular to the coronal plane, the CT images stacked together could be regarded as a discrete 3D model. Based on the labeled reference center CT image and the center point of the labeled nodule rectangle, we can obtain the 3D samples by clipping the stacked CT images with a pixel block size of 32 × 32 × 7.

### 2.2. Network Architecture

In this study, the lack of raw information is one major issue need to be resolved. On the one hand, the total number of nodule samples in the dataset is small compared with typical sample number of deep learning dataset. On the other hand, the resolution of the sample images acquired from LDCT is low. The classification performance of our network lies on the solution of the raw information inadequacy. Therefore, it is necessary to design a network with strong feature extraction ability.

#### 2.2.1. Average-Max Attention Residual Learning Block

**Figure 2:**
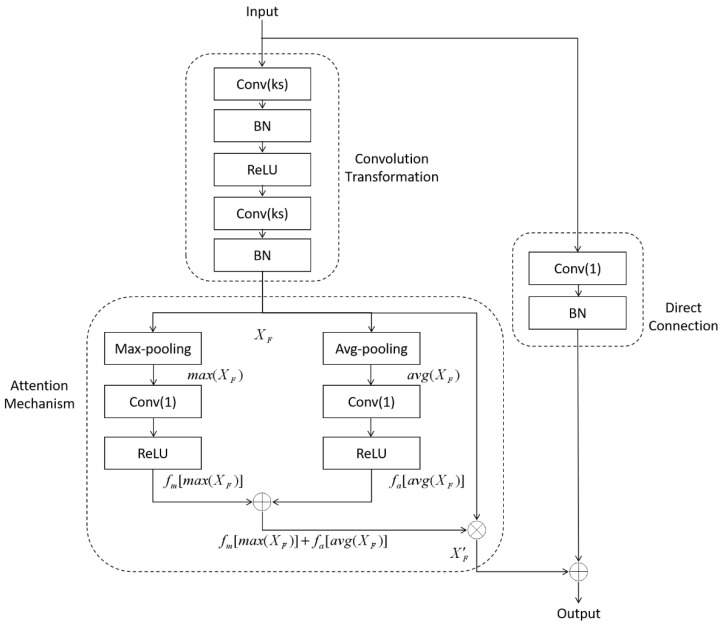
Average-Max Attention Residual Learning Block.

We construct a residual learning block with an attention mechanism [21]. This learning block uses a residual network block [22] as our basic structure because it can overcome the over fitting problem caused by having too many training parameters. In view of the fact that the size of lung nodules and the location of effective image information varies widely in different samples, we apply attention mechanism to the residual network block. The new learning block is called Average-Max Attention Residual Learning Block and the structure is shown in Figure 2.

Symbol ⊕ denotes the addition on the feature maps. Symbol ⊗ denotes multiplication on the feature maps. Conv(·) represents the convolution layer with its kernel size (·). For example, Conv(1) represents the convolution layer, the kernel size is 1 × 1 when in 2D network and 1 × 1 × 1 when in 3D network. BN and ReLU represent batch normalization and activate function ReLU, respectively.

There are three parts of the learning block: convolution transformation, direct connection and attention mechanism. The convolution transformation is a typical feature extraction unit with two layers of convolution *Conv* (*ks*). As for the direct connection part, we choose a convolution layer *Conv* (1). Along with the convolution layer is a batch normalization layer. The purpose of direct connection part is to concatenate feature map with different size.

The attention mechanism part is mainly composed of two parts, namely the mean attention and the maximum attention. In each channel, after the feature map is input to the block, the feature map is further extracted by convolution transformation. We denote the feature map after convolution transformation as *X_F_*. Then the weight of the feature map *X_F_* is calculated by the mean and maximum values of the feature map. This step is accomplished by a max-pooling layer and an average-pooling layer in practice. After the pooling layers, the maps become *max* (*X_F_*) and *avg* (*X_F_*), respectively. Following up each map is a convolution layer *Conv* (1) combined with activation function ReLU. The calculation of convolution and activation is denoted by function f(·). In this case, the maps become *f_m_*[*max* (*X_F_*)] and *f_a_*[*avg*(*X_F_*)]. We consider the combination of *f_m_* [*max* (*X_F_*)] and *f_a_* [*avg* (*X_F_*)] as the weight value of feature map *X_F_*. Finally, by multiplying feature map *X_F_* with weight value *f_m_*[*max* (*X_F_*)] + *f_a_*[*avg*(*X_F_*)], we realize the attention mechanism of our block. We sort out the above calculation and we can get the final calculation formula of the attention mechanism:

(1) $${X^{\prime }}_{F}=\{f_{m}[max(X_{F})]+f_{a}[avg(X_{F})]\}\cdot X_{F}$$

The output feature map ${X^{\prime }}_{F}$ is added with the feature map of initial input feature map as the output feature map of the learning block. Then it is provided to the subsequent part of the network for training and learning.

The proposed learning block integrate residual network and attention transformation. The convolution transformation part of residual network is used to extract the feature map of lung nodules. Attention transformation operates on the extracted feature map so that the important feature channels are set to have higher weights. This construction enables the network to learn important features in lung nodules. The direct connection part of the network can avoid the degradation of network and can better combine the sample features extracted from former layers.

#### 2.2.2. Multi-Level and Multi-Feature Hybrid Learning Network

**Figure 3:**
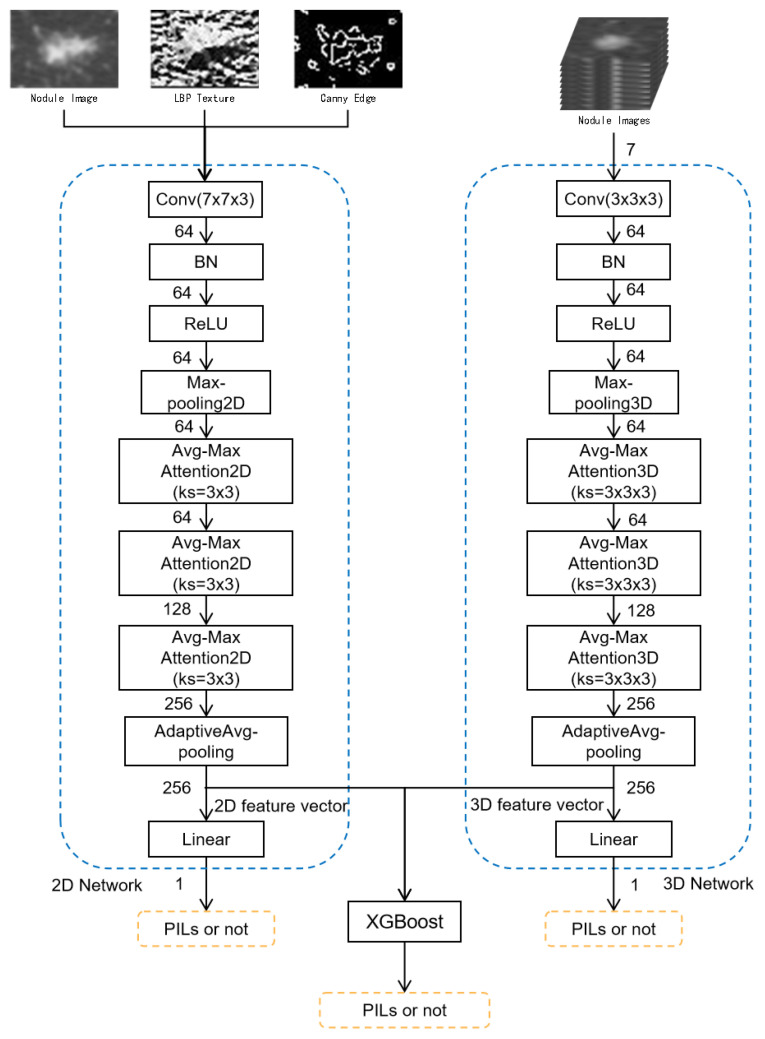
Structure of the Multi-level and Multi-feature Hybrid Learning Network.

The architecture of our network is shown in Figure 3. The kernel size of each layer is shown in the block diagram within the bracket. The number on the arrow between layers is the channel number. Our network consists of three modules: 2D network, 3D network and XGBoost hybrid learning. 2D samples and 3D samples are input to the 2D network and 3D network, respectively. Based on the labels of the samples, the 2D network and 3D network are trained separately. After training, the feature vectors of the 2D network and 3D network are concatenated together as the input of XGBoost to be further trained.

It can be observed from Figure 3 that the structure of the 2D network and 3D network are similar. The main difference between the 2D network and 3D network is the kernel size of the layers. Therefore, we explain the structure of the network once in the following part. First there is a convolution layer along with batch normalization (BN) and activate function ReLU. This step is the initial data preprocessing. The obtained initial feature maps are further transformed using max-pooling to remove redundant information. Then, we thoroughly extract the features with three layers of Avg-Max Attention learning block connected in series. Finally, the learned feature map in each channel is computed to be one feature value by adaptive average pooling and we use a fully connected layer to compute the result of the classification.

In training, the 2D network and 3D network are also trained separately. The optimizer of the training process is stochastic gradient descent (SGD) [27] with momentum to accelerate the convergence speed of training. We select the binary cross entropy function as the loss function.

The L2 regularization term is added into the loss function to prevent the over fitting of the training process. The final loss function is:

(2) $$Loss_{BCE}=\frac{1}{n_{SAM}}\sum_{i}^{n_{SAM}}(L(i)\cdot \ln(\hat{L}(i))+(1-L(i))\ln(1-\hat{L}(i)))+\alpha_{L2}{\sum \left\|w_{M}\right\|}_{2}^{2}$$

where $n_{SAM}$ is the total number of the samples. L(i) is the true label of sample i and $\hat{L}(i)$ is the predictive value of sample i computed by the network. $\alpha_{L2}{\sum \left\|w_{M}\right\|}_{2}^{2}$ is the L2 regularization term with $\alpha_{L2}$ as the coefficient of the term.

After the two networks are trained, we concatenate the final feature vector from 2D network and 3D network. The concatenated vector is expressed as:

(3) $$v_{fused}=[v_{3D}(1),v_{3D}(2),\cdots ,v_{3D}(256),v_{2D}(1),v_{2D}(2),\cdots ,v_{2D}(256)]$$

where $v_{3D}(i)$ denotes the vector on the *i*th channel of the 3D feature vector. $v_{2D}(i)$ denotes the vector on the *i*th channel of the 2D feature vector.

We take the concatenated vector of the samples as a new dataset. And we use XGBoost algorithm to classify the new dataset. XGBoost algorithm is an excellent decision tree classification algorithm. It predicts the actual value of the sample by means of residual learning. This algorithm has proved its efficiency and accuracy during years of application. The original CT images are too complicated to learned by XGBoost. Therefore, we preliminarily extract the features of the original CT images with the help of neural network. This formulation makes the expression of features more concise and more adapted to the computation of the XGBoost algorithm.

As for our dataset, XGBoost generates different trees to fit the classification model. With a given feature vector $v_{fused}$, each tree in the whole tree family finds its corresponding leaf node, i.e., predictive value, based on the value of $v_{fused}$. Then the algorithm adds all the predictive value given by all the trees in the tree family to output the classification result. The objective optimization function of the algorithm is:

(4) $$Obj_{XGB}=\sum_{i=1}^{n_{SAM}}DS(L(i),\hat{L}(i))+\Omega_{XGB}+const$$

where $DS(L(i),\hat{L}(i))$ is the quadratic loss function of the sample true value L(i) and predictive value $\hat{L}(i)$. $\Omega_{XGB}$ is a regularization function representing the complexity of the model tree. *cosnt* is a constant term in the model.

## 3. Experiments and Results

The experiments of our network are conducted on a machine with Intel Core i9–9900 CPU, NVIDIA GeForce RTX 2080ti GPU, 32 GB memory and the Ubuntu 18.04 operating system. Our code is based on Python 3.7 and Pytorch deep learning framework.

### 3.1. Data Preparation

Among all the 1752 cases collected, there are 676 PIL nodule cases and 1076 other cases. We regard the PIL nodule cases as the positive samples of the dataset and other cases as the negative samples of the dataset. In the test set, there are 100 positive samples and 100 negative samples. In the training set, there are 576 positive samples and 976 negative samples.

Due to the limited number of samples, the training process implies many uncertainties. Therefore, we expand our dataset with 1752 samples into 92,928 samples using traditional offline augmentation method such as random image translation, rotation, and flip. The number of positive samples are expanded to 46,080 and the number of negative samples are expanded into 46,848.

In order to improve the efficiency and accuracy of our network, we employ an online augmentation algorithm as well. There are two popular augmentation methods: Cutout and CutMix [28]. The cutout method cuts out an area and fills up the cut area with 0s which are a common value in CT images. This may weaken the attention and locating ability. It is not thus appropriate for our dataset. CutMix cuts a random part of a sample, and then replaces this part with the corresponding region of another sample from the dataset. The label of the new sample is a soft label generated by splicing and is calculated according to the proportion of the two samples. Therefore, the CutMix method is more suitable for our network to focus on the vital features of the images.

### 3.2. Model Evaluation

In order to better express the classification ability of the model, accuracy, sensitivity, specificity, F1 score, ROC curve and AUC are used to measure the classification performance. These indexes are commonly used for evaluation in the field of binary classification. Accuracy represents the overall diagnostic accuracy. Sensitivity represents the percentage of people having the disease who are correctly identified as having the disease. The higher sensitivity number means that the probability of missed detection is low. Specificity represents the percentage of healthy people who are correctly identified as healthy. The higher specificity number means that the probability of false detection is low. F1 score represents the weighted combination of accuracy and recall rate. ROC (receiver operating characteristic) reflects the perceptibility to the same signal stimulus. AUC is the area under the ROC curve.

### 3.3. Training Preparation

In the experiments, the Kaiming method [29] is used to initialize parameters in the network. The probability of data being augmented online by Cutmix is 50%. The initial learning rate of the 3D network is 0.02. The initial learning rate of the 2D network is 0.004. We apply attenuation mechanism in the changing of learning rate. That is, every several training step, the learning rate decay a certain scale. The decay rate in our experiments is set to be 0.9 for every 10 epochs. For example, the learning rate of the 3D network becomes 0.018 on epoch 10. The coefficient *α_L_*_2_ of the L2 regularization term is 1 × 10^−6^ in the two networks. After many experimental attempts, we set the parameters of XGBoost as follows: The maximum depth of the tree is 8. The learning rate is 0.02. The maximum number of iterations is 60.

### 3.4. Results

We run experiments using the above settings. Firstly, the performance comparison of the 2D network itself, the 3D network itself and XGBoost with feature fusion is carried out. The results are shown in Table 1. The confusion matrix of our XGBoost model is provided in Table 2. We discuss the result of confusion matrix (possible reasons of misclassification) in Section 4. It can be observed that XGBoost can get better results in the classification task after fusing the feature learned from 2D network and 3D network.

Secondly, under the same parameter setting of training process, the performance comparison of using CutMix augmentation and without using CutMix augmentation is carried out. The result is shown in Table 3. Although there is a slight decrease in the specificity, the accuracy and sensitivity is improved by using CutMix augmentation.

**Figure 4:**
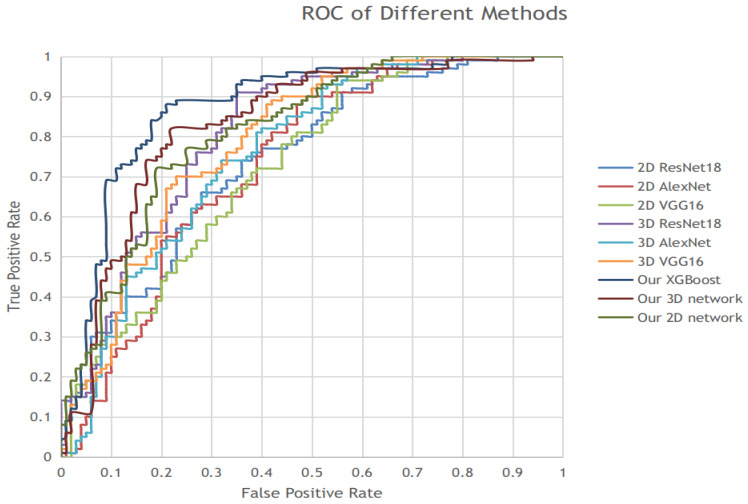
ROC of Different Methods.

Thirdly, we run many state-of-the-art networks on our dataset to compare the performances. Three trending networks are selected: VGG16, AlexNet and ResNet18. These three networks have proven their accuracy in the binary classification task. Because the samples in our dataset involving 2D samples and 3D samples, we run their 2D version and 3D version, respectively. The results of the experiment are shown in Table 4 and Figure 4. The results suggests that our model has achieved better performance not only after learning with XGBoost, but also before fusion learning as in the stage of 2D network and 3D network.

## 4. Discussion

Based on the results presented in Section 3, we can conclude the novel part of our model. Our efforts are mainly focused on the processing of the data and the designing of network architecture.

In the data preparation and augmentation stage, we formulate a variety of methods to achieve better performance of the model. The initial data is divided into 2D samples and 3D samples so that the network can learn features in different dimensions. The 2D samples are composed of three different types of feature map to make information more abundant. As for augmenting the dataset, we make use of the traditional offline augmentation method and CutMix online augmentation. The results in Table 3 prove the effectiveness of the augmentation.

The architecture of the network in our model is designed meticulously. We first design an average-max attention residual learning block based on the basic structure of ResNet. The attention mechanism in the block can “pay more attention” on the critical channel of feature maps, leading to better classification results when the features of the initial maps are sparse. The results are presented in Table 4 and Figure 4 and show its advantage compared to traditional ResNet. In addition, we fuse the final feature vector obtained from the 2D network and 3D network by using XGBoost. This formulation further improves the performance of our model by a large scale, as shown in Table 1.

Although our model has achieved better performance than other models, it has not reached a really outstanding classification result. The ideas we used in our model are fundamental. There are many cutting edge ideas may improve the performance significantly.

**Figure 5:**
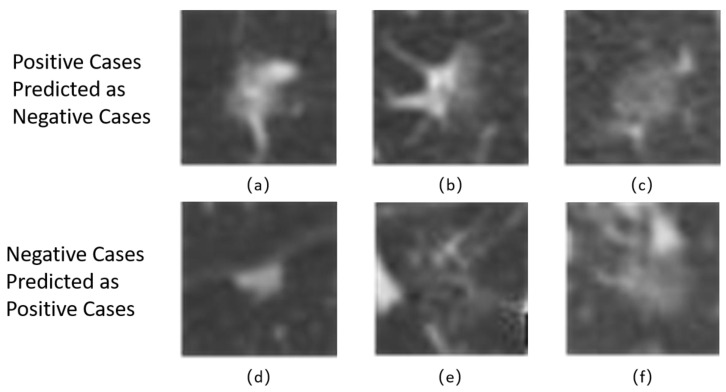
Typical cases that are classified wrongly.

Based on the confusion matrix result listed in Table 2, there are still some cases that are classified wrongly. In Figure 5 we show some typical misclassification cases (PILs classified as others and other cases classified as PILs) and give some possible reasons why they are misclassified. The positive cases (PILs) predicted as negative cases (others) occurs under two circumstances, when there are capillaries or bronchi adjacent to the nodule (Figure 5a,b) and when the nodules have regular shape with uniform density (Figure 5c). The former (Figure 5a,b) may be wrongly classified as IAs because it exhibit uneven image intensity which is common in IAs. The latter (Figure 5c) may be wrongly classified as IAs because the size of the nodule is large which is also common in IAs. Vice versa, the reasons of negative cases predicted as positive cases are similar. They either have small size (Figure 5d) or they show very few distinct differences, which makes them be classified as PILs.

We believe that the threats to validity is low. Although the total amount of cases in our dataset is not very large, the cases we collected have significant representativeness. Many cases are confusing for the doctors and can only be diagnosed by biopsy. These cases should cover most of the cases encountered in clinical diagnosis.

## 5. Conclusions

In this paper, we present a neural network learning model to classify the PIL nodules out from a composite LDCT dataset. The network is based on multi-dimension (2D and 3D) networks and multi information learning. In the training process, we employ CutMix to further augment the training data. The main highlight of our network lies in the designed “Avg-Max Attention Residual Learning Block” and XGBoost integrated learning. The comparison results between our network with the learning block and other networks without the learning block have proved the effectiveness of the learning block both in 2D and 3D networks than other network structures. After fusing the learning result of the 2D network and 3D network using XGBoost, the classification result improved significantly. The results and discussion presented in Section 3 and Section 4 show that the performance of our network is promising. The results predicted by our model can be regarded as supplementary information in routine clinical screening for doctors to make diagnoses.

## Figures and Tables

**Table 1:** The performance of different model in our architecture.

Models	Accuracy	Sensitivity	Specificity	F1 Score	AUC
2D Network	0.75	0.79	0.71	0.76	0.81
3D Network	0.785	0.82	0.75	0.79	0.83
XGBoost	0.83	0.86	0.8	0.83	0.88

**Table 2:** The confusion matrix of our XGBoost model.

		**Predicted Class**	
		**Positive**	**Negative**
Actual Class	Positive	86	14
	Negative	20	80

**Table 3:** The performance of the XGBoost before and after using CutMix augmentation.

Models	Accuracy	Sensitivity	Specificity	F1 Score	AUC
Without CutMix	0.81	0.81	0.81	0.81	0.87
With CutMix	0.83	0.86	0.8	0.83	0.88

**Table 4:** The performance of our model and other state-of-art models.

Models	Accuracy	Sensitivity	Specificity	F1 Score	AUC
2D VGG16	0.655	0.66	0.66	0.65	0.72
2D AlexNet	0.675	0.63	0.69	0.66	0.73
2D ResNet18	0.68	0.66	0.70	0.67	0.73
Our 2D network	0.75	0.79	0.71	0.76	0.81
3D VGG16	0.72	0.82	0.62	0.74	0.79
3D AlexNet	0.69	0.77	0.61	0.71	0.76
3D ResNet18	0.75	0.81	0.69	0.76	0.81
Our 3D Network	0.785	0.82	0.75	0.79	0.83
XGBoost	0.83	0.86	0.8	0.83	0.88
